# Improvement in Microstructure and Properties of 304 Steel Wire Arc Additive Manufacturing by the Micro-Control Deposition Trajectory

**DOI:** 10.3390/ma17051170

**Published:** 2024-03-02

**Authors:** Huijing Zhang, Weihang Liu, Xiaohui Zhao, Xinlong Zhang, Chao Chen

**Affiliations:** 1 Key Laboratory of Advanced Structural Materials, Ministry of Education, School of Materials Science and Engineering, Jilin Provincial Key Laboratory of Advanced Materials Processing and Application for Rail Transit, Changchun University of Technology, Changchun 130012, China; 2 Key Laboratory of Automobile Materials, School of Materials Science and Engineering, Jilin University, Changchun 130025, China; 3 College of Mechanical and Electrical Engineering, Northeast Forestry University, Harbin 150040, China

**Keywords:** 304 stainless steel, micro-control deposition trajectory, swing arc, microstructure

## Abstract

In this study, the GMAW welding torch was controlled by a stepping motor to achieve a periodic swing. By controlling the swing speed, a micro-variable deposition path was obtained, which was called the micro-control deposition trajectory. The influence of the micro-control deposition trajectory on the arc characteristics, microstructure, and mechanical properties of 304 steel wire arc additive manufacturing was studied. The results showed that the micro-control deposition process was affected by the swing arc and the deposition trajectory and that the arc force was dispersed over the whole deposition layer, which effectively reduced the welding heat input. However, the arc centrifugal force increased with the increase in the swing speed, which easily caused instability of the arc and large spatter. Compared with common thin-walled deposition, the deposition width of micro-control thin-walled deposition components was increased. In addition, the swinging arc had a certain stirring effect on the molten pool, which was conducive to the escape of the molten pool gas and refinement of the microstructure. Below, the interface of the deposition layer, the microstructure of the common thin-walled deposition components, and the micro-control thin-walled deposition components were composed of lathy ferrite and austenite. Compared with the common deposition, when the swing speed increased to 800 °/s, the microstructure consisted of vermicular ferrite and austenite. The tensile strength and elongation of the micro-control thin-walled deposition components are higher than those of the common thin-walled deposition components. The tensile fracture mechanism of the common thin-walled deposition components and the micro-control thin-walled deposition components was the ductile fracture mechanism.

## 1. Introduction

Additive manufacturing is a revolutionary breakthrough processing technology. It is a dimension reduction processing method that cuts a complex three-dimensional part model into a two-dimensional model with a certain thickness. These 2D slices are then stacked layer by layer until the three-dimensional part is obtained. Compared with traditional manufacturing methods (casting, forging, etc.), additive manufacturing can reduce complicated steps such as casting models, shorten the manufacturing time and cycle of products, and improve manufacturing efficiency and material utilization [1,2,3,4,5]. The wire arc additive manufacturing (WAAM) process uses the arc as the heat source to melt the wire and adopts the layer-by-layer cladding principle to continuously deposit the additive components. Compared with the laser additive manufacturing process and electron beam additive manufacturing process, WAAM has the advantages of rapid deposition speed, high material utilization rate, strong flexibility, low technical cost, simple equipment, and the ability to form complex large components [6,7,8]. WAAM has become one of the important choices for the forming and manufacturing of complex metal components. However, the WAAW process will produce uneven heat input and high-temperature liquid metal, making the additive components subjected to repeated thermal cycling [9]. These problems result in the grain coarsening and seriously restrict the mechanical properties of WAAM components.

Researchers mainly focus on grain refinement and property improvement by optimizing process parameters or adding external assistance [10,11,12,13,14]. Anirban Bhattacharya et al. investigated the effect of heat input on AISI 304 stainless steel welded joints and found that high heat input and low cooling rate lead to grain size increase, while high heat input and rapid cooling rate could inhibit grain growth and refine grains [15]. Arc oscillation or swing has the effect of dispersing arc force, stirring molten pool, promoting molten metal flow, and refining grain. Diego Raimundi Corra et al. proposed a method of applying a magnetic field in tungsten arc additive manufacturing to achieve arc oscillation. They found that the vibration mode and frequency had a significant impact on arc characteristics and molten pool behavior [16]. Wang Lei et al. proposed a laser oscillation welding method and compared the effects of oscillation modes (transverse, longitudinal, and circular) on the microstructure and properties of aluminum alloys [17]. The results pointed out that the laser beam oscillating pattern had a stirring effect on the molten pool, resulting in the formation of equiaxed crystals in the fusion zone of the aluminum alloy-welded joint. Compared with no vibration, the elongation of the specimen in circular vibration mode increased by 38%. Yuan, T. et al. pointed out that transverse arc oscillation reduces the temperature gradient along the welding direction, which is conducive to the decomposition of dendrites and the formation of equiaxial crystals [18]. 

During the arc welding process, arc swing has the effect of dispersing arc force, stirring molten pool, promoting molten metal flow, and refining grain. Yang, L. and Guo, N. et al. found that rotating arc narrow gap horizontal welding not only reduces effective welding heat input but also can disperse the arc force and improve the metal transfer [19,20]. Yong Chen et al. found that when the welding torch oscillated along the thickness of the welding plate, the reciprocating upward-rotating arc promoted the stirring of the molten metal and the stirring effect strengthened the flow of the molten metal, which has a positive effect on the weld grain refinement [21]. From the above research, it can be found that the microstructure and properties of welded joints can be significantly improved by using swinging or oscillating arcs. However, there is little research on the use of swinging or oscillating arcs to improve the microstructure and properties of additive components.

In this paper, the GMAW was used to prepare 304 stainless steel components. The periodic swing of the GMAW welding torch was obtained by using a stepper motor. By controlling the swing period, a micro-variable deposition path is obtained, which is called the micro-control deposition trajectory. Based on WAAM, this paper studies the effect of the micro-control deposition trajectory on the arc characteristics, microstructure, and properties of 304 stainless steel thin-walled deposition components. Firstly, the influence of micro-control trajectory on the arc characteristics is researched by the high-speed camera. Then, the microstructure and mechanical properties of 304 stainless steel deposition components are studied in detail by the optical microscope (OM), tensile tests, and scanning electron microscope (SEM). Finally, the mechanism of micro-control trajectory arc additive manufacturing is clarified. Through the above research, a new high-quality arc additive manufacturing method will be developed in the field of arc additive manufacturing.

## 2. Materials and Methods

**Figure 1:**
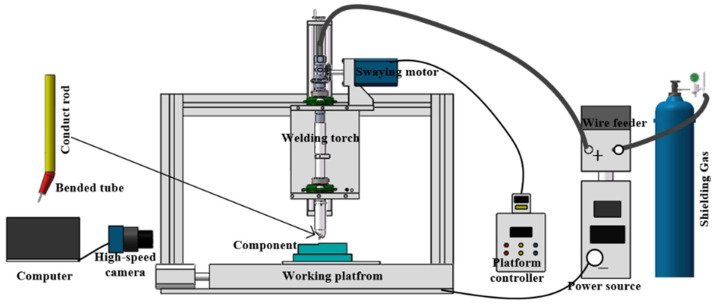
Schematic diagram of the experimental equipment.

Figure 1 shows the schematic diagram of the experimental equipment. As shown in Figure 1, the self-designed micro-controlled deposition WAAM equipment (Kemppi FASTMIG350, Lahti, Finland)was used in this paper, which is composed of a welding power source, a three-axis motion platform, and a micro-controlled trajectory welding torch. Before the deposition process, the oxide film of the base material surface was removed by the angle grinder. The welding wire used in the present study was 304 stainless steel wire with dimensions of 1.0 mm and 304 stainless steel with a thickness of 15 mm was selected as the base material. The chemical compositions of 304 stainless steel wire and 304 stainless steel are shown in Table 1. The shielding flow rate was 20 L/min (99.99% Ar). The image of arc behavior was obtained by the high-speed camera. The frame rate of 1000 fps and exposure time of 100 μs were adopted. 

**Figure 2:**
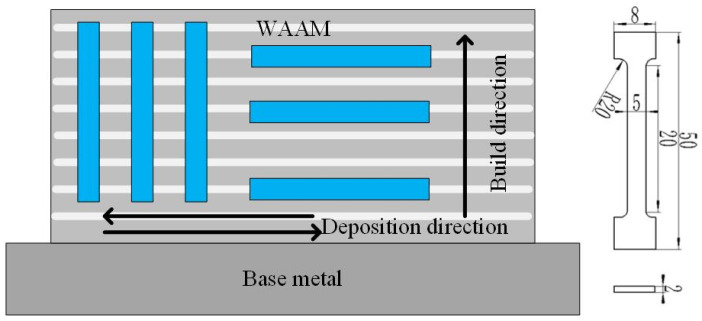
Schematic of sampling preparation methods and dimension of the tensile specimens (in mm).

In this paper, the GMAW welding torch was controlled by a stepping motor to achieve a periodic swing. By controlling the swing cycle, the micro-change deposition path was obtained, which was called the micro-control deposition trajectory. The influence of the micro-control deposition trajectory on the arc characteristics, microstructure, and mechanical properties of 304 steel wire arc additive manufacturing was studied. During the deposition process, the wire feeding speed was 8 mm/s, the bending radius of the contact tip was 6 mm, the arc swing angle was 60°, and the welding speed was 4.5 m/min. The reciprocating swing of the welding gun was taken as one cycle. The swing speed and frequency are shown in Table 2. The swinging speed is the angular velocity of the arc rotating around the center of the conductive nozzle. The metallographic specimens with the dimension of 5 mm × 20 mm × 30 mm were prepared by electric spark wire cutting technology. Then, the metallographic specimens were ground and polished. Finally, after etching for 20 s, the microstructure of the metallographic specimens was observed by the Zeiss Axio Imager A1m optical microscope (OM). The metallographic corrosion liquid was 50 mL HCl, 10 mL HNO_3_, 10 g FeCl_3_, and 100 mL H_2_O. A tensile test was carried out on the electronic universal testing machine at room temperature. The tensile rate of 2 mm/s was adopted. Two types of tensile specimens, vertical plane and horizontal plane, were tested, respectively. Figure 2 shows the schematic of the sampling preparation methods and dimension of the tensile specimens.

## 3. Results and Discussions

### 3.1. Arc Characteristics

**Figure 3:**
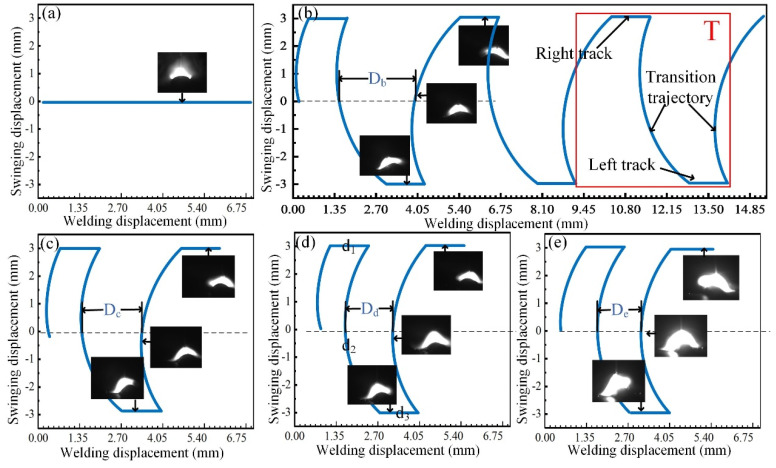
Arc characteristics of the different deposition trajectories: (**a**) S−0; (**b**) S−200; (**c**) S−400; (**d**) S−600; and (**e**) S−800.

The deposition trajectory is the result of the joint action of swing speed and welding speed. Figure 3 shows the arc shapes under the different micro-control deposition trajectories. Figure 3a shows the arc shapes under the common deposition trajectory. The arc appears directly above the deposition layer, exerting a vertical downward force that leads to the highest temperature at the center of the deposition layer. Figure 3b–e shows the arc shapes under micro-control deposition trajectories. D represents the distance between two adjacent arcs that move towards the center of the thin-walled deposition components. The period of deposition trajectory, as depicted in the red box in Figure 3b, can be divided into three stages, namely, the right trajectory, the transition trajectory, and the left trajectory. The arc shapes of both right and left trajectories are symmetrical. The arc movement mode lasts for 0.3 s on the left side and transitions to the right side for 0.3 s, repeating this motion consistently. At the swing speeds of 200 °/s, 400 °/s, 600 °/s, and 800 °/s, the arc tilts from the sides towards the sedimentary layer, ensuring even heating throughout the entire deposition layer. During this process, increasing swing speed leads to a higher instantaneous deposition speed while reducing the welding heat input and dispersing arc force [22,23]. With the increase in swing speed, the arc size increases gradually. The maximum arc size is observed when the swing speed reaches 800 °/s (Figure 3e). This occurs due to an increase in arc centrifugal force as swing speed increases, which results in arc instability [24].

**Figure 4:**
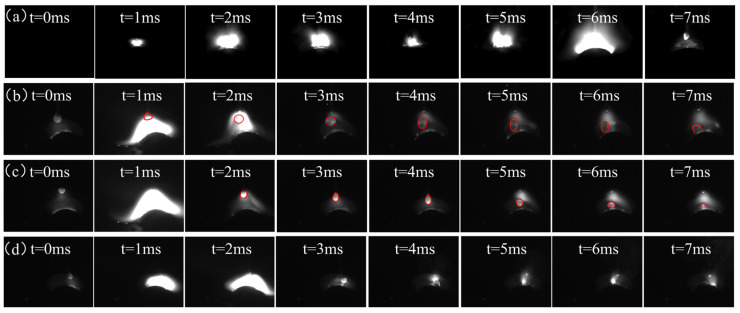
Arc and droplet morphology: (**a**) S−0; (**b**) left trajectory of S−600; (**c**) transition trajectory of S−600; and (**d**) right trajectory of S−600.

The evolution of microstructure is determined by the solidification process of the molten pool. During this process, there is an accompanied epitaxial growth of grains, a change in the temperature gradient in the molten pool, and different crystal growth rates. These conditions are influenced by factors such as arc striking position, welding conditions, welding process, etc. [25,26]. Figure 4a shows the arc and droplet morphology of the common deposition. The droplet enters the molten pool from the top of the deposition layer and the molten pool is subjected to both vertical downward arc pressure and droplet impact. The transition form of the droplet is the short circuit transition. Figure 4b shows the arc and droplet morphology on the left trajectory of the S−600 specimen. The droplet morphologies are marked with a red circle. The centrifugal force generated by the arc swing breaks the original arc balance, resulting in dispersed arc force and a large arc area. The arc frequency of micro-control deposition is lower than that of the common deposition. During micro-control deposition, droplets enter the molten pool from the upper left side of the deposition layer, which enhances the fluidity of the molten pool in the horizontal direction and plays a stirring effect in the molten pool. At this stage, the transition form of the droplet is considered to be in the droplet transition [27,28,29]. Figure 4c,d shows the arc and droplet morphology in the transition trajectory and right trajectory of the S−600 specimen, respectively. Figure 4c,d shows the arc and droplet morphology in the transition trajectory and right trajectory of the S−600 specimen, respectively. The arc and droplet morphology on the left trajectory are similar to that of the right trajectory. The arc size on the right trajectory and left trajectory of the S−600 specimen is significantly larger than that of the S−0 specimen.

### 3.2. Appearance and Microstructure of Thin-Walled Component

**Figure 5:**
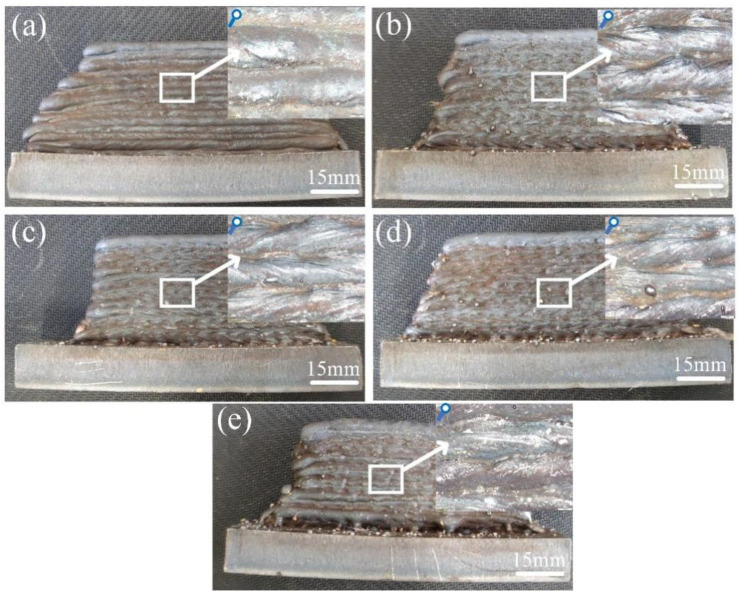
Appearance of thin-walled deposition components: (**a**) S−0; (**b**) S−200; (**c**) S−400; (**d**) S−600; and (**e**) S−800.

In this section, the influence of the swing speed of micro-control deposition on the thin-walled deposition components’ appearance is studied (Figure 5). The upper right corner is the partially enlarged images of the surface of the thin-walled deposition components. As shown in Figure 5a, there is no obvious splash on the surface of the common thin-walled deposition component. The local convex phenomenon on the surface of the common deposition layer is caused by the molten pool solidification under the effect of surface tension during deposition. The droplet spatter increases with the increase in the swing speed, which is caused by the centrifugal force generated by the swing affecting the arc stability (Figure 5b–e). In the partially enlarged image, it can be seen that the winding lines appear on the surface of the micro-control deposition layer, which is caused by the swinging arc stirring the molten pool and promoting the molten pool flow [23]. 

**Figure 6:**
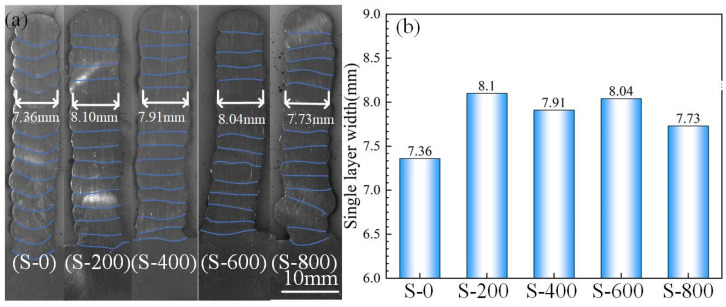
Cross-section of thin-walled deposition components with different deposition trajectories: (**a**) cross-section morphology and (**b**) the single layer width.

Figure 6 shows the cross-section morphology and the single-layer width of thin-walled deposition components with different deposition trajectories. There are no obvious defects in the cross section of the thin-walled components under the different deposition trajectories (Figure 6). As shown in Figure 6a, the shape of the fusion line of the common thin-walled components is a concave arc. The width of the single deposition layer is 7.36 mm (Figure 6b), while the fusion line of micro-control thin-walled deposition components is horizontal and the penetration is relatively uniform. The width of the single deposition layer of the micro-control thin-walled deposition components is significantly increased compared with that of the common thin-walled deposition components. When the swing speed is 200 °/s, 400°/s, 600 °/s, and 800 °/s, the width of the single deposition layer is about 8.1 mm, 7.91 mm, 8.04 mm, and 7.73 mm, respectively (Figure 6b). This is because in the process of micro-control trajectory deposition, the arc uniformly acts on the whole deposition layer, so that the deposition layer has a more uniform penetration. At the same time, the heat distribution area increases and the energy density decreases when the arc swings. During the deposition process, a small amount of melting occurred in the previous deposition layer and the temperature of the molten pool decreased [16]. However, the excessive swing rate affects the stability of the arc, making the deposition layer uneven.

**Figure 7:**
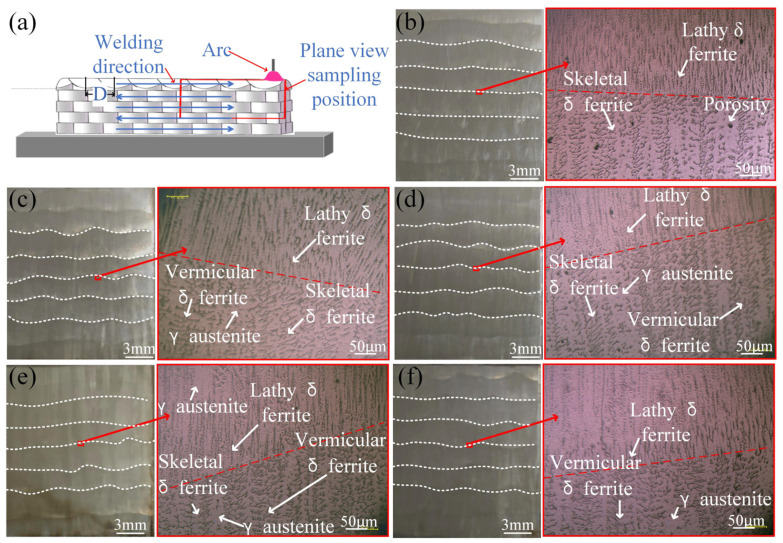
Sampling diagram of the metallographic specimens (**a**) and microstructure of thin-walled components with different swing speeds: (**b**) S−0; (**c**) S−200; (**d**) S−400; (**e**) S−600; and (**f**) S−800.

Figure 7 shows the sampling diagram of the metallographic specimens and the microstructure of thin-walled deposition components. The metallographic specimens were taken from the upper right center of the thin-walled deposition components, as shown in the red box of Figure 7a. As shown in Figure 7b–f, the left side is a macro metallographic view of the thin-walled deposition components under different swing speeds and the right side is an enlarged view of the interface microstructure of the deposition layer. The interface of the deposition layer of the common thin-walled deposition components is a straight line, while that of the micro-control thin-walled deposition components is a staggered wavy line. With the increase in the swing speed, the distance of D also decreases. The arc is mainly struck from both sides and the proportion of the transition phase in the cycle decreases. The arc on both sides remelts the molten pool in the transition phase, making the wavy lines at the interface of the deposition layer denser.

Austenite (γ) and multiform ferrite (δ) distribute in the deposition layer. Above the interface of the deposition layer of the common thin-walled deposition components and the micro-control thin-walled deposition components, the microstructure is composed of lathy ferrite and austenite. Below the interface of the deposition layer, the microstructure of the common thin-walled deposition components and the micro-control thin-walled deposition components is composed of ferrite and austenite. However, the morphology of ferrite is quite different. As shown in Figure 7b, below the interface of the deposition layer, the microstructure of the common thin-walled deposition components mainly consists of skeletal ferrite and austenite. At the same time, there are many micropores in the material. Below the interface of the deposition layer, the microstructure of the S−200 specimen is vermicular ferrite, skeletal ferrite, and austenite (Figure 7c). As shown in Figure 7c,d, when the swing speed is 400 °/s and 600 °/s, the same microstructure appears below the interface of the deposition layer. When the swing speed is 800 °/s, the microstructure is vermicular ferrite and austenite. The micro-controlled arc can fully stir the molten pool, leading to the fragmentation of dendrites and the formation of various forms of ferrite. With the increase in the swing speed, the arc movement cycle reduces and the skeletal ferrite transforms into a mixture of vermicular and skeletal ferrite and finally into a single vermicular ferrite. This indicates that the increase in arc oscillating velocity is beneficial to the cooling of the deposition layer. The type of the ferrite transformation is due to the cooling rate [28]. In FA solidification mode, when the cooling rate is low, the vermicular ferrite and skeletal ferrite appear on the austenitic matrix. This is a result of the advance in the austenite consuming the ferrite until the ferrite is fully enriched in ferrite-promoting elements (such as chromium). When the cooling rate is high, vermicular ferrite appears on the austenitic matrix. This is due to the fact that during the phase transition, the cooling rate is fast and the element diffusion is limited. Deposition cracks mainly occur along the dendrite grain boundary. Some ferrites can improve crack resistance and reduce the solidification cracks generation [30]. Micro-control deposition changes the position of arc and droplet deposition, making the deposition layer more evenly heated. During the common deposition, ferrite grows perpendicular to the fusion line at the interface of the deposition layer. However, the interface of the micro-control deposition layer is a wavy line, which makes the ferrite not only grow along the vertical direction but also inclines the staggered growth. This also results in the improvement in mechanical properties of micro-control thin-walled deposition components.

### 3.3. Mechanical Behavior of the Thin-Walled Component

**Figure 8:**
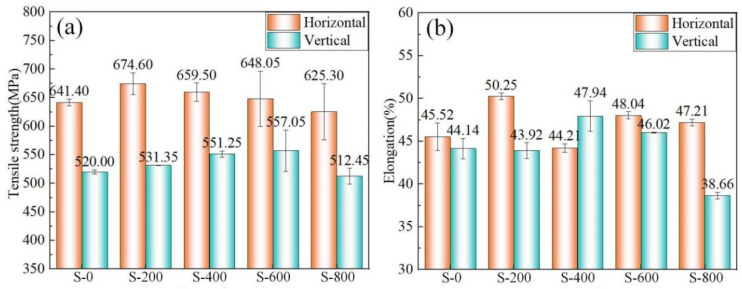
Tensile test results: (**a**) tensile strength and (**b**) elongation.

Figure 8 shows the horizontal and vertical tensile strength and elongation of thin-walled deposition components. The horizontal tensile strength of the common thin-walled deposition components is 641.4 MPa and the horizontal elongation is 45.52%. The vertical tensile strength of the common thin-walled deposition components is 520 MPa and the vertical elongation is 44.13%. The tensile strength and elongation of micro-control thin-walled deposition components are higher than those of the common thin-walled deposition components. Compared with the common thin-walled deposition components, when the swing speed is 400 °/s, the horizontal and vertical tensile strength of the micro-control thin-walled deposition components is increased by 2% and 6%, respectively. As the swing increases, the tensile strength decreases. When the swing speed is 800 °/s, the tensile strength and elongation of micro-control thin-walled deposition components are lower than those of common deposition thin-walled components. This is consistent with the corresponding results in Figure 3, that is, when the oscillation frequency increases, the centrifugal force increases, thus affecting the stability of the arc and the properties. The refined microstructure can improve the tensile strength, especially the vertical tensile strength of the micro-control thin-walled deposition components. The difference between the horizontal and vertical tensile strength of the micro-control thin-walled deposition components (S−400, S−600, and S−800) is reduced compared with that of the common thin-walled deposition components (S−0), which proves that the micro-control deposition trajectory method can effectively promote the isotropy of the grain.

**Figure 9:**
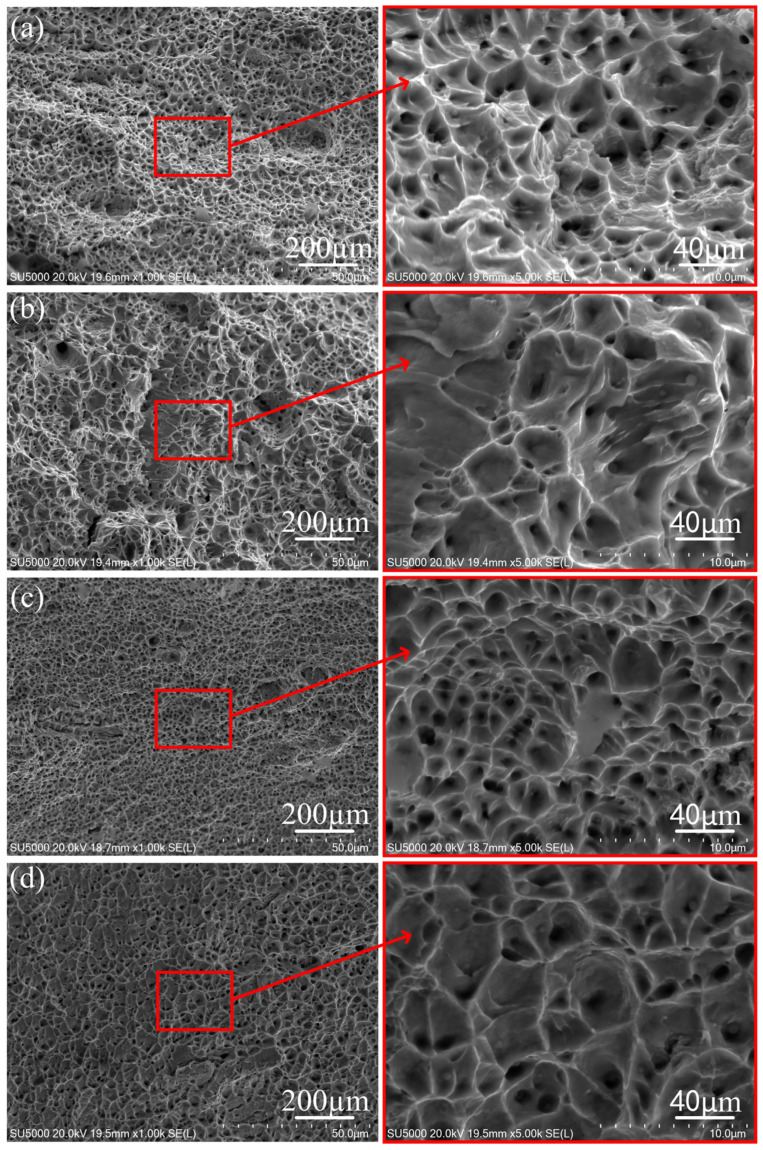
Fracture morphology: (**a**) horizontal tensile specimen of S−0; (**b**) vertical tensile specimen of the S−0; (**c**) horizontal tensile specimen of S−600; and (**d**) vertical tensile specimen of S−600.

Figure 9 shows the fracture morphology of the horizontal and vertical tensile specimens of the S−0 specimen and the S−600 specimen. As shown in Figure 9, the tensile fracture morphology of all specimens is a ductile fracture. The fracture surface is composed of a large number of equiaxed ductile dimples. The size of the ductile dimples of the vertical tensile specimen is significantly larger than that of the horizontal tensile specimen, indicating that the plasticity of the deposition components is better in the vertical direction. In the local magnification of Figure 9b, there are a large number of dimples, tearing edges, and pores in the fracture. These pores are formed due to the inability of gas to escape from the molten pool. These pores are also consistent with those in Figure 7b, reducing the tensile properties of the thin-walled deposition components. As shown in Figure 9c,d, pores cannot be found in the fracture of micro-control thin-walled deposition components, which indicates that the oscillating arc stirs the molten pool, promotes the flow of the molten pool, facilitates the escape of gas, and reduces the pore defects. Compared with the S−0 specimen, the dimples on the tensile fracture surface of the S−600 specimen are finer and denser, indicating that the tension strength of the S−600 specimen is better than that of the S−0 specimen.

## 4. Conclusions

1. The micro-control deposition trajectory technology disperses the arc force and evenly heats the deposited layer. When the swing rate is too high, the centrifugal force of the arc is too large, resulting in an unstable arc and large splash. In the common deposition, the transition form of the droplet is the short circuit transition. The molten pool is affected by vertically downward arc pressure and droplet impact, resulting in a deeper middle and the shallower sides of the molten pool, while the micro-control deposition trajectory is affected by the deposition path and swing speed. The transition form of the droplet is the droplet transition. The micro-control deposition trajectory technology has achieved a relatively uniform melting depth and increased the stirring effect of the molten pool;
2. There is no obvious defect in the micro-control thin-walled deposition components. The cross-section fusion line of the common thin-walled deposition components is a concave arc. The transverse fusion line of the micro-control thin-walled deposition components is horizontal and the melting depth is more uniform;
3. With the increase in the swing rate, the proportion of transition trajectory stages in the period decreases. Below the interface of the deposition layer, the microstructure of the common thin-walled deposition components and the micro-control thin-walled deposition components is composed of lathy ferrite and austenite. Compared with the common deposition, when the swing speed increased to 800 °/s, the microstructure consisted of vermicular ferrite and austenite;
4. The tensile strength and elongation of the micro-control thin-walled deposition components are higher than those of the common thin-walled deposition components. The tensile fracture mechanism of the common deposition and the micro-control deposition is the ductile fracture mechanism. There are a lot of dimples and tear edges in the fracture. Compared with the micro-control thin-walled deposition components, there are porosity defects in the common thin-walled deposition components.

In the future, the molten pool flow, temperature, and stress behavior in the process of the micro-control deposition trajectory will be researched to discuss the microstructure evolution and mechanical properties of deposition components. In addition, the use of the electromagnetic micro-control method and mechanical arm micro-control method to further improve micro-control deposition technology will also be the focus of future research.

## Figures and Tables

**Table 1:** Chemical compositions of the base material and steel wire (wt%).

Materials	C	Si	Mn	P	S	Ni	Cr
Base material	≤0.08	≤1.00	≤2.00	≤0.035	0.03	8.00–11.00	17.00–19.00
Steel wire	0.05	0.44	1.43	0.03	0.01	8.08	18.08

**Table 2:** Deposition process parameters.

Specimen	Swing Speed (°/s)	Frequency (Hz)	D (mm)
S−0	0	0	0
S−200	200	0.83	2.685
S−400	400	1.11	2.100
S−600	600	1.33	1.637
S−800	800	1.48	1.522

## Data Availability

All relevant data are within the paper.
